# Supplementary material for: Time to phlebitis onsite and its predictors among admitted patients with peripheral intravenous cannulas at Debre Markos Comprehensive Specialized Hospital, Northwest Ethiopia, 2024/ 2025: A prospective follow-up study
Source: PLoS One. 2025 Nov 4;20(11):e0336176. doi: 10.1371/journal.pone.0336176 (PMC12585079; doi:10.1371/journal.pone.0336176)
Supplement: Attachment — Submitted filename: Comments.docx [file pone.0336176.s001.docx]

**Abstract section**

**Introduction**

1. The introduction effectively highlights the significance of phlebitis from peripheral intravenous cannulation. However, it would benefit from explicitly defining the outcome variable(s), such as the incidence of phlebitis and the time to onset after cannula insertion. Clarifying these variables will provide a clearer focus for the study and enhance the readers' understanding of its objectives.

**Method**

**Why the median time is used instead of mean? Is there any reason?**

**Result section**

**Comment:**

1. **Under the socio demographic section**

It contains variable out of sociodemographic variable on line 312-314, I noted that out of the 168 female participants, 11 (6.55%) were found to be pregnant, and 45 (26.79%) reported using contraceptives, including 35 (20.80%) specifically using Depo-Provera. It would be beneficial for the authors to clarify whether these findings fall under the sociodemographic variables or if they should be categorized as health-related variables. Typically, sociodemographic variables include age, education level, income, and marital status, while pregnancy status and contraceptive use may be better classified as reproductive health indicators. To enhance the clarity of the results section, I suggest the authors explicitly define their variable categories. This will help readers understand the context of the findings and their implications for the study. Additionally, presenting these variables separately may provide deeper insights

**Main body of the manuscript**

**Comments on the VIP Scoring System:**

1. **Range of Scores**: The VIP scoring system starts at zero and goes directly to two. This means that there is no defined "core" score of one, which could represent a milder or early stage of phlebitis. The jump from zero to two may limit the system's sensitivity in detecting very early signs of phlebitis.
2. **Clinical Implications**: By not having a score of one, the system may overlook subtle symptoms that could indicate the onset of phlebitis. This could impact early intervention strategies, as healthcare providers might not recognize early-stage phlebitis that warrants monitoring or treatment.
3. **Potential for Improvement**: It may be beneficial for the VIP scoring system to consider including a score of one. This could enhance the tool’s sensitivity and provide a more nuanced understanding of phlebitis progression.
4. **Consistency in Clinical Practice**: The current structure may lead to inconsistencies in clinical assessments if practitioners do not recognize the absence of a milder score. Clarifying this in training or documentation could help mitigate confusion.

### Generally In summary, the lack of a core score of one in the VIP scoring system may limit its effectiveness in early detection of phlebitis. Consideration of this gap could lead to improvements in patient assessment and care. So if there is any justification for this please include

**Discussion section**

**It is well written and compared with the existing literature , however the most important thing is the implication of the study with regard to the patient, health care professional as well as the policy maker should be implicitly discussed , simplify comparing the results has no important so please see it and discuss the pertinent findings giving emphasis for implication of the findings**
