# Supplementary material for: Time to phlebitis onsite and its predictors among admitted patients with peripheral intravenous cannulas at Debre Markos Comprehensive Specialized Hospital, Northwest Ethiopia, 2024/ 2025: A prospective follow-up study
Source: PLoS One. 2025 Nov 4;20(11):e0336176. doi: 10.1371/journal.pone.0336176 (PMC12585079; doi:10.1371/journal.pone.0336176)
Supplement: Attachment — Submitted filename: Response to Reviewers.docx [file pone.0336176.s002.docx]

We would like to say thank you (academic editor) for fast response for our submission.

Also, we would like to thank the reviewers for sacrificing your valuable time and providing constructive comments on this study. We have made all corrections in the main document, highlighted in yellow for reviewer one, purple color for reviewer two and green for editor based on your feedback in the main document named revised manuscript with change.

Contents

[Editor comments	1](#_Toc209609667)

[Reviewer's One Questions and Comments:	2](#_Toc209609668)

[Reviewer #2 comments and Q	12](#_Toc209609669)

# Editor comments

1. Please ensure that your manuscript meets PLOS ONE's style requirements, including those for file naming.

**Response**

We used PLOS ONE's style requirements, including those for file naming.

2. In the ethics statement in the Methods, you have specified that verbal consent was obtained. Please provide additional details regarding how this consent was documented and witnessed, and state whether this was approved by the IRB

**Response**

Thank you, we modified this in the revised manuscript with retract changed that highlighted by green color.

3. Please include captions for your Supporting Information files at the end of your manuscript, and update any in-text citations to match accordingly.

**Response**

We changed the supporting information by narration, which was highlighted in green color in the revised manuscript with retract changed.

4. If the reviewer comments include a recommendation to cite specific previously published works, please review and evaluate these publications to determine whether they are relevant and should be cited. There is no requirement to cite these works unless the editor has indicated otherwise. 
**Response**

We carefully examined the paper suggested by the reviewers. After evaluating their relevance to our study, we incorporated the works that were directly pertinent and strengthened our paper. For those that were less important to our paper, we did not add them.

5. Please review your reference list to ensure that it is complete and correct. If you have cited papers that have been retracted, please include the rationale for doing so in the manuscript text, or remove these references and replace them with relevant current references. Any changes to the reference list should be mentioned in the rebuttal letter that accompanies your revised manuscript. If you need to cite a retracted article, indicate the article’s retracted status in the References list and also include a citation and full reference for the retraction notice.

**Response**

- Thank you for your valuable comments. We carefully reviewed our reference list to ensure accuracy and completeness. During this process, we identified and replaced some older references with more recent and relevant papers. None of the cited articles are retracted; therefore, no retraction notices were required. All changes to the reference list have been reflected in the revised manuscript.

# Reviewer's One Questions and Comments:

We would like to thank the reviewer for sacrificing your valuable time and providing constructive comments on this study. We have made all corrections in the main document, highlighted in yellow, based on your feedback. However, corrections highlighted in pink were made for Reviewer Two. Thank you both

**Reviewer’s Q:**

The manuscript titled “Time to develop phlebitis and its predictors among admitted patients with peripheral intravenous cannulas at Debre Markos Comprehensive Specialized Hospital, Northwest Ethiopia, 2024/2025: a prospective follow-up study” addresses an important topic in patient safety and intravenous therapy management. The study provides valuable data on the incidence and predictors of phlebitis in an Ethiopian hospital setting, which is a relatively under-researched area. Overall, the manuscript is informative and well-structured. However, there are several points regarding clarity, methodology, data presentation, discussion, and ethical considerations that should be addressed to strengthen the manuscript.

**Author's Response:**

Thank you very much for your constructive and insightful feedback on this paper. We greatly appreciate your recognition to the importance of our research work. We carefully reviewed all the points you mentioned regarding to the clarity, methodology, data presentation, discussion, and

We carefully reviewed all the points you raised regarding clarity, methodology, data presentation, discussion, and ethical considerations, and we have made the necessary corrections and improvements throughout the manuscript.

**Reviewer’s Q:**

Abstract
Background: Some sentences in the background (line 33-37) repeat ideas about phlebitis being a clinical problem without focusing directly on the research gap. So please write the background to highlight the knowledge gap in Ethiopia more clearly.
Methods: It is good that statistical methods (Kaplan–Meier, Cox regression) are mentioned, but they could briefly state why survival analysis was chosen (time-to-event nature).
Result: the results are well presented with cumulative incidence and median time to event. It may help readers if confidence intervals are also provided for incidence and survival estimates, not just hazard ratios.
Conclusion: The conclusion is consistent with the results. However, the authors might phrase recommendations for clinical practice or implications more directly (e.g., “Clinicians should monitor patients with 18-gauge cannulas or receiving vancomycin more closely…”).
Please consider adding a brief statement on the major limitation of the study in the abstract (e.g., sample size, study design), as this is important for balance and clarity.

**Author's Response:**

Thank you for your valuable comment. We did an update on the abstract thanks

**Reviewer’s Q:**

Introduction
The manuscript currently separates Background and Objectives into two distinct sections. In journal article format, it is preferable to combine these into a single Introduction section. The introduction should begin with a concise overview of the clinical relevance of peripheral intravenous cannula (PIVC) and phlebitis. Summarize global and local incidence rates, highlighting the gap in Ethiopian data. End with a clear statement of the study objectives.

**Author's Response:**

Thanks, we corrected it.

**Reviewer’s Q:**

Introduction
The background is informative but overly detailed in places, which may obscure the main research gap. For instance, lines 62–64 include a long sentence with multiple procedures listed; this could be simplified for readability.

**Author's Response:**

lines 62–64:- *While it aids in medication administration, fluid infusion, blood transfusion, and supports numerous therapeutic and diagnostic procedures like hemodialysis, parenteral nutrition, and contrast agent delivery, patient may experience complications like phlebitis*

changed by

*While this procedure is essential for giving medications, fluid, blood transfusions, and supporting treatments and diagnostic procedures like hemodialysis, parenteral nutrition, and contrast studies, it can also cause complications, the most frequent of which is phlebitis.*

This keeps the contrast clear and makes it flow as a single complete sentence. We would like to appreciate the concern.

**Reviewer’s Q:**
Line 61: “Each year, over one billion intravenous cannulas are placed for patients in hospitals patients globally” → appears redundant (“patients” twice).

**Author's Response:** Thanks, corrected.

**Reviewer’s Q:**
Line 76: “It may be sue to fail to adhere” → should be “due to failure to adhere.”

**Author's Response:**

Thanks, corrected.

**Reviewer’s Q:**
Ensure terminology is consistent (e.g., “predictors” vs. “predicators” in line 106).

**Author's Response:**

Thanks, corrected.
**Reviewer’s Q:**

The background cites global incidence (3–70%), with examples from multiple countries, which is strong. However, it may help to briefly note why Ethiopia shows such a high rate (70% in Gondar hospital). Is it due to methodological differences, healthcare infrastructure, or clinical practices?

**Author's Response:**

Thank you for the insightful comment. The high incidence reported in Gondar Hospital (70%) may be attributed to factors such as differences in study methodology, variations in healthcare infrastructure, and specific clinical practices, including frequent use of peripheral intravenous cannulae and differences in infection prevention measures. To maintain clarity and conciseness in the background, we focused on reporting the incidence, while these potential contributing factors are discussed in detail in the discussion section.

**Reviewer’s Q:**
Line 70: Suggest rephrasing “Among mechanical factors, the size of the cannula and the site of insertion play a significant role” → could be “Mechanical factors such as cannula size and site of insertion significantly influence risk.”

**Author's Response:**

Several factors can contribute for occurrence of phlebitis. These are mechanical, chemical, and bacterial causes. Mechanical factors, such as cannula size and insertion sit, can irritate the vein wall and trigger inflammation. Chemical factors also contribute, particularly the type of solution and the infusion speed, with the faster rates increasing the likelihood of vein irritation. In addition, bacterial factors play a critical role, as improper sterile technique during cannula insertion can enter bacteria into the bloodstream, resulting to infection.

**Reviewer’s Q:**
Formatting: Citations are in parentheses but inconsistent spacing (e.g., “clinical setting(1)” → should be “clinical setting (1)”).

**Author's Response:**

- Thank you.

**Reviewer’s Q:**

Methods
The methodology is described in detail, which is commendable. However, the section is very long and could benefit from more concise phrasing. Some repetition (e.g., in sample size determination and cannula definitions) could be reduced for clarity.

**Author's Response:**

We used more concise phrase in the main document particularly in method section. Fore example, cannula definition was done as follow

Cannula size was recorded according to the outer diameter of the needle, measured in French (Fr) units, with corresponding gauge and color codes: 16-gauge (gray, 5Fr), 18-gauge (green, 3.8Fr), 20-gauge (pink, 2.7Fr), 22-gauge (blue, 2.2Fr),24 gauge (yellow, 1.7Fr).

**Reviewer’s Q:**
Study area and period: The study area is limited to medical, surgical, and orthopedic wards. However, important clinical areas such as maternal, or ICU wards were not included. This restriction may affect the generalizability of the findings, as the risk of phlebitis and related predictors could differ across different patient populations. I recommend that the authors acknowledge this limitation, particularly in the abstract and discussion sections.

**Author's Response:**

- Thank you for the constructive feedback. We acknowledge it in the limitation section

**Reviewer’s Q:**
Study design and population: The design (prospective follow-up) is appropriate for the research objective.

**Author's Response:**

- Okay, thank you, we wrote based on this.

**Reviewer’s Q:**
Inclusion and exclusion criteria are well stated. However, justification for excluding patients with altered consciousness could be clarified (e.g., inability to provide consent, difficulty in observing outcomes).

**Author's Response:**

- Thank you for the constructive feedback. Patients with altered consciousness were excluded primarily because they were unable to provide informed consent and their condition made it difficult to reliably assess and observe the study outcomes. This exclusion was necessary to ensure both ethical considerations and the accuracy of data collection

**Reviewer’s Q:**
Sample size and sampling technique: The proportional allocation and systematic sampling are appropriate, though the potential limitation of systematic sampling (risk of hidden periodicity bias) should be acknowledged.

**Author's Response:**

- **Thanks, we incorporated it as follow in the limitation section**

Lastly, there may be a potential of risk of hidden periodicity bias when systematic sampling applied.

**Reviewer’s Q:**
Data collection: It would help to specify whether inter-rater reliability was checked among data collectors when assessing phlebitis scores, to strengthen methodological rigor.

**Author's Response:**

- Be honest we did not conduct inter-rate reliability. This may enhance methodological rigor. What we did was when the data collector decided the score the supervisor cross check again and if there was discrepancy, it was solved with a discussion with other staffs who are working in the hospital based on the criteria.

**Reviewer’s Q:**
Data quality assurance: Translation and back-translation are appropriate, but a note on whether content validity or reliability testing of the questionnaire was performed would strengthen this section.

**Author's Response:**

- Thank you, we checked the reliability of the questionnaire and modification done the main document

**Reviewer’s Q:**
Data management and analysis: Testing of proportional hazards assumptions and checking for multicollinearity are strengths. However, the authors should mention how missing data (if any) were handled.

**Author's Response:**

- Thank you for your comment whereas, we were not faced this challenge. The nature of the data collection was not faced us for this challenge.

**Reviewer’s Q:**
Ethical consideration: The manuscript mentions that ethical approval was obtained, but the reference number or formal approval letter is not provided. For transparency and compliance with journal standards, it is recommended to include the ethics committee approval reference number and, if available, the date of approval. This ensures readers can verify that the study was conducted in accordance with ethical guidelines.
**Author's Response:**

- You are correct we did modification on it, thanks. Also, we attached the letter as well.

Ethical clearance was obtained from Debre Markos University College of Medicine and Health Sciences Research Ethics and Approval Committee**, with reference number RCSTTD/395/01/17, dated 01/11/2024**.

**Reviewer’s Q:**
Result
Socio-demographic characteristics: The reporting of contraceptive use could be clarified further; Is the denominator all female participants (168), or only non-pregnant ones?

**Author's Response:**

- It is only non-pregnant females, and we tried to correct in the main document as follow;

Additionally, 45(26.8%) **of the non-pregnant female participants** reporte**d** using contraceptives, with 35 (20.8%) specifically using Depo-Provera

**Reviewer’s Q:**
Clinical, Infusion, cannula-related characteristics: Nearly three-fourths of the study participants were admitted with a single diagnosis” – It would be clearer to provide the exact number and percentage instead of a vague phrase (“nearly three-fourths”).

**Author's Response:**

We incorporated the exact number with its percentage.

**Reviewer’s Q:**
The phrase “three-fourths of the participants had a normal BMI” is too approximate. Giving exact numbers and percentages would be more precise.

**Author's Response:**

We modified it as follow;

Regarding body mass index, 223(59.9%) of the participants had a normal body mass index, whereas 26 participants (7.0%) could not be categorized due to general body swelling, pregnancy, or disability

**Reviewer’s Q:**
Treatment Data (IV fluids, drugs, injections, blood transfusion): The flow is a bit disorganized. It mixes different interventions without grouping them logically. Consider restructuring: first fluids/drugs, then injection frequency, then transfusion.

**Author's Response:**

**Thanks, we did correction as follow;** Out of the total participants, 345 (92.7%) received intravenous drugs, and 192 participants (51.6%) received two or fewer injections per day. Whereas, 155 (41.7%) participants received intravenous fluids, and 37(10.0%) participants received blood transfusions.

**Reviewer’s Q:**
Almost half patients (51.61%) received two or fewer injections per daily” → needs correction for grammar: “…per day.

**Author's Response:**

- Thanks, we did correction.

**Reviewer’s Q:**
Again, percentages like 29.03%, 35.19%, 41.67%, etc. are overly precise. Rounding to one decimal place (e.g., 29.0%, 35.2%, 41.7%) is more standard in reporting.
**Author's Response:**

- Thanks, we did correction.

**Reviewer’s Q:**

Predictors of phlebitis: Use either “Cox proportional hazards regression” or “Cox regression” consistently. Mixing terms (“Cox proportional hazards model”, “Cox proportional regression”) may confuse readers.

**Author's Response:**

We made it uniform/ consistent through the document to avoid confusion on readers

**Reviewer’s Q:**
Discussion
This is a strong discussion with thorough literature engagement and plausible explanations, but it is too wordy, repetitive, and detail-heavy. It would benefit from reorganization, clearer writing, and more emphasis on implications.
The discussion provides a strong comparison with previous studies; however, it would benefit from additional justification for the high incidence of phlebitis in your setting. Beyond methodological and demographic explanations, systemic factors in Ethiopia (such as limited resources, high nurse-to-patient ratios, lack of standardized IV protocols, and socio-economic challenges) may have contributed.

**Author's Response:**

We appreciate for your fruitful comments. We tried to update the whole discussion part by using more recent published papers with their explanation to the difference exist**.**

**Reviewer’s Q:**
Recommendations
For health organizations: Consider rephrasing “ensure availability of alternative medications to Vancomycin and KCl” alternatives may not always exist; instead, focus on safe administration practices, staff training, and supply of appropriate equipment.

**Author's Response:**

You are right, thank you, we modified it based on your recommendation in main document.

**Reviewer’s Q:**
Limitation of the study
Expand slightly to include other study limitations, e.g., single-center design, observational nature, and potential for residual confounding.

**Author's Response:** we tried to included all that can be as limitation for this study. However, we did VIF to minimize residual confounding. Thanks

# Reviewer #2 comments and Q

The author sincerely thanks the reviewer for your valuable time, constructive feedback, and insightful comments, which have significantly contributed to improving the quality and clarity of this study
**Reviewer’s Q**

Abstract Section
Define outcome variables like incidence of phlebitis and time to onset for clarity.

**Authors response**

Thank you for the comment. We agree on the importance of defining outcome variables. However, to minimize word count and adhere to the journal’s abstract guidelines, we provided detailed definitions of the outcome variables such as incidence of phlebitis and time to onset in the main document rather than in the abstract.
Methods
Clarify the choice of median over mean.
**Authors response**

Thank you for the insightful comment. We used the median instead of the mean because our data were not normally distributed, and the median provides a more accurate measure of central tendency in the presence of skewed data
**Reviewer’s Q**

Results Section
Specify if pregnancy status and contraceptive use are sociodemographic or health-related variables for better categorization.

**Authors response**

We included in one table is due to minimize the number of tables.
however, to undress your concern we modified in some extent in heading thanks.

**Reviewer’s Q**

Main Body
VIP Scoring System: The lack of a score of one may limit early detection of phlebitis. Consider adding this score to improve sensitivity and clinical assessments.

**Authors response**

Thank you for your valuable comment. We acknowledge the concern regarding the absence of a score of one in our operational definition. However, the Venous International Phlebitis (VIP) scoring system we used is a standardized and validated tool, in which a score of two or higher is considered indicative of phlebitis. Modifying this tool by redefining the cut-off point could compromise its reliability, validity, and comparability with other studies. For this reason, we adhered strictly to the original scoring system without altering its operational definition.

**Reviewer’s Q**

Discussion Section
Emphasize the study's implications for patients, healthcare professionals, and policymakers rather than just comparing findings.

**Authors response**

Thank you for your comment. we incorporate all the study’s implication in the recommendation section.
